# Self-rated joint hypermobility: the five-part questionnaire evaluated in a Swedish non-clinical adult population

**DOI:** 10.1186/s12891-020-3067-1

**Published:** 2020-03-17

**Authors:** Martin Glans, Mats B. Humble, Marie Elwin, Susanne Bejerot

**Affiliations:** 1 Faculty of Medicine and Health, University Health Care Research Centre, Örebro University, Örebro, Sweden; 2 School of Medical Sciences, Örebro University, Örebro, SE-70182 Örebro, Sweden; 3 Department of Clinical Neuroscience, Karolinska Institutet, Stockholm, Sweden

**Keywords:** Hypermobility, Joint instability, Surveys and questionnaires, Validation, Translation, Diagnostic self evaluation

## Abstract

**Background:**

The conventional way to identify generalised joint hypermobility is by a physical examination according to the Beighton Score. However, a physical examination is time-consuming in clinical practise and may be unfeasible in population-based studies. The self-assessment five-part questionnaire on hypermobility (5PQ) offers a more practicable way to identify GJH. The aim of this study was to test validity and reliability of the five-part questionnaire on hypermobility (5PQ) translated into Swedish on a non-clinical adult population.

**Methods:**

A structured procedure was used for the translation of the 5PQ into Swedish. The Beighton Score was used as reference standard for generalised joint hypermobility. Test-retest reliability was tested in a separate group who filled in the questionnaire twice with a ten-week interval. Participants consisted of a convenience sample recruited in Stockholm, Sweden (2017).

**Results:**

A total of 328 participants were included in the study, 297 participants in the validity group and 31 participants in the reliability group. When evaluated against a present Beighton Score with an age-dependent cut-off, the Swedish 5PQ attained a sensitivity of 91%, a specificity of 75% and an area under the curve of 0.87. The Swedish 5PQ showed substantial to almost perfect test-retest reliability.

**Conclusions:**

The Swedish 5PQ is a valid and reliable instrument to screen for or to identify generalised joint hypermobility.

## Background

Generalised joint hypermobility (GJH) is a term used to describe the ability to extend synovial joints beyond their normal limits [1]. GJH can be asymptomatic or symptomatic. When symptoms are present, it might be part of generalised hypermobility spectrum disorder or a heritable disorder of connective tissue, such as Ehlers-Danlos syndrome [1]. GJH seems to occur in 10–20% in the general population although its presence is influenced by age, gender and ethnicity [2]. The conventional way to identify GJH is by a physical examination according to the Beighton Score (BS) [3]. However, a physical examination is time consuming in clinical practise and may be unfeasible in population-based studies. The self-assessment five-part questionnaire on hypermobility (5PQ) [4] offers a more practicable way to identify GJH. The 5PQ was initially developed in English to identify people with symptomatic hypermobility however, it has also been shown to be helpful for identifying asymptomatic GJH in population based studies [5]. To our knowledge the only published translation is into Brazilian-Portuguese [6].

### The aim of this study

The aim of this study was to test validity and reliability of the 5PQ translated into Swedish on a normal adult population.

## Methods

### Study population

A total of 297 participants were recruited for the validity test. This was based on a sample size calculation [7] with an expected sensitivity and specificity of 80% respectively; an expected prevalence of GJH of 20% and a desired absolute precision of 10%. Participants consisted of a convenience sample recruited in Stockholm, Sweden (2017) from various settings: a university campus (*n* = 168), a community health centre (*n* = 112) and the marketing department at the national public TV broadcaster (*n* = 17). An additional sample of 31 university students was recruited for reliability tests.

Inclusion criteria were age 18–65 years and a full understanding of the Swedish language. Exclusion criteria were a physical condition hampering the BS assessment or having any missing data on the Swedish 5PQ. Participants were informed that the aim was to collect data on joint mobility in an adult population.

### Measures

#### The reference standard -the Beighton score (BS)

The BS measures hypermobility at nine joints. Traditionally, hypermobility was suggested if ≥4 oof the 9 joints were hypermobile in an adult [2]. According to recent recommendations [8], for the diagnosis of hypermobile Ehlers-Danlos syndrome, the BS cut-off for GJH in pubertal men and women up to 50 years is ≥5/9 while the cut-off of ≥4/9 is used for adults 50 years and above. Furthermore, the inclusion of historical information on hypermobility by the means of the 5PQ is suggested for individuals with a physical condition hampering the BS assessment. If the BS is 1 point below the cut-off and the 5PQ is positive, then a diagnosis of GJH still can be made. Since the goal of our study was to validate the 5PQ we did not want to include it in the reference test.

#### The index test -the five-part questionnaire on hypermobility (5PQ)

The 5PQ consists of five items. Each endorsement yields one point and two or more points suggests GJH [4] (Table 1). The 5PQ was developed using the BS as a reference standard test and sensitivity and specificity have been reported between 71–84% and 77–89% respectively [4, 6]. The Brazilian-Portuguese validation of the 5PQ applied a present BS of ≥4/9 as a cut-off for GJH [6]. The original 5PQ authors did not clearly specify whether historical hypermobility was included in their validating BS tests [4].

**Table 1:** The Five-Part Questionnaire (5PQ) [4] for defining Generalised Joint Hypermobility

1. Can you now (or could you ever) place your hands flat on the floor without bending your knees?	
2. Can you now (or could you ever) bend your thumb to touch your forearm?	
3. As a child did you amuse your friends by contorting your body into strange shapes OR could you do the splits?	
4. As a child or teenager did your shoulder or kneecap dislocate on more than one occasion?	
5. Do you consider yourself double-jointed?	
Endorsement of two or more questions suggests generalised joint hypermobility.	

### Procedures

#### Translation of the questionnaire into Swedish

The original authors granted permission to translate the 5PQ. Forward translation was done by a health professional, familiar with terminology of the area, native in Swedish and with a good command of English (the first author, MG). A panel including the last author SB (medical doctor, with a wide experience in validating rating scales); the first author MG (medical doctor) and a layman native in English and fluent in Swedish discussed the preliminary Swedish version with regard to language and cultural applicability before a consensual version was achieved. Two professional translators, native in English and unaware of the original version, back-translated the preliminary adaptation. The back-translated versions were compared to the original questionnaire whereby a final version was agreed upon. The final version was communicated to the original authors. The translation process is shown in Supplementary Table S1, Additional file 1. The Swedish 5PQ Screen print-out version with instructions to the clinician is available as supportive information in Additional file 3.

When the original version of the 5PQ is compared to our back-translated versions (Supplementary Table S1, Additional file 1), we consider item 3 “As a child did you amuse your friends by contorting your body into strange shapes OR could you do the splits?” and item 5 “Do you consider yourself double-jointed?” to differ the most from the original version. Regarding item 3, the words “amuse”, “contort” and “shapes” were back-translated to “entertain”, “twist” and “positions”, which we consider being correct synonyms. Regarding item 5, “double-jointed” was back-translated to “clearly hypermobile in your joints” and “to have joint hypermobility”. There is no Swedish expression equivalent to “double-jointed”, and the Swedish phrasing was considered as informal. We believe that the differences in wording were not such as to affect the overall understanding or interpretability of the Swedish version of the 5PQ.

### Data collection

Participants completed a survey including the Swedish 5PQ and demographic data, followed by a clinical examination with the BS. A goniometer was used to assess the fifth finger, elbow and knee (standing). The examiner was a trained physician, blinded to the results of the Swedish 5PQ (the first author, MG). The subjects did not warm up prior to the examination. Any physical condition hampering the examination was recorded. For reliability testing, the Swedish 5PQ was administered and re-administered after 10 weeks to a subgroup of 31 subjects. The reliability group did not undergo a physical examination.

### Statistics

Statistical analyses were primarily conducted in IBM SPSS statistics version 23. MedCalc version 18.5 was used to calculate 95% confidence intervals on sensitivity, specificity, positive predictive value (PPV) and negative predictive value (NPV).

### Validity

The validity of the Swedish 5PQ was tested against two different criteria of the reference standard test: 1) a present BS with an age-dependent cut-off of ≥5/9 for individuals 18–50 years and ≥ 4/9 for individuals > 50 years, according to the updated recommendations and 2) a present BS with the traditional cut-off of ≥4/9. Post hoc analyses were performed on additional cut-off scores on the reference standard test. Non-parametric assumptions about the distribution were adopted in the construction of the receiver operator characteristic (ROC) curves. Area under the curve (AUC) was interpreted as no discrimination (0–0.5), poor (0.5–0.7), fair (0.7–0.8), good (0.8–0.9) or excellent (0.9–1.0) accuracy [9]. There are no agreed standards by which to judge sensitivity and specificity, rather the optimal balance between sensitivity and specificity depends on the purpose of the test. Generally, a screening test should prioritise a high sensitivity, while a follow-up confirmatory test should be highly specific [10]. Predictive values are, on the other hand, not only dependent on sensitivity and specificity but also on the prevalence of the disease in the studied population. Accordingly, in populations with a high prevalence of the disease, the PPV will be higher than in populations or settings where the disease is rare [10].

### Test-retest reliability

Test-retest reliability for each item and for the total-score of the Swedish 5PQ was examined by Cohen’s un-weighted kappa and intra-class correlation coefficient (ICC), respectively. Cohen’s kappa was interpreted as slight (0–0.2), fair (0.2–0.4), moderate (0.4–0.6), substantial (0.6–0.8) and almost perfect (0.8–1.0) agreement [10]. ICC estimates were calculated based on a single-measurement, absolute agreement, two-way mixed-effect model. ICC was interpreted as poor (0–0.5), moderate (0.5–0.75), good (0.75–0.9) or excellent (0.9–1.0), [11].

### Item specific analyses

The distribution of responses for each item on the 5PQ was determined. Odds ratios (OR) for GJH for each item were calculated by 2 × 2 crosstabulation tables and analysed with Pearson’s Chi-square test. Fisher’s exact test was used when expected value of a cell was less than 5. *P* values reported are 2-sided.

## Results

### Characteristics of the study population

**Fig. 1:**
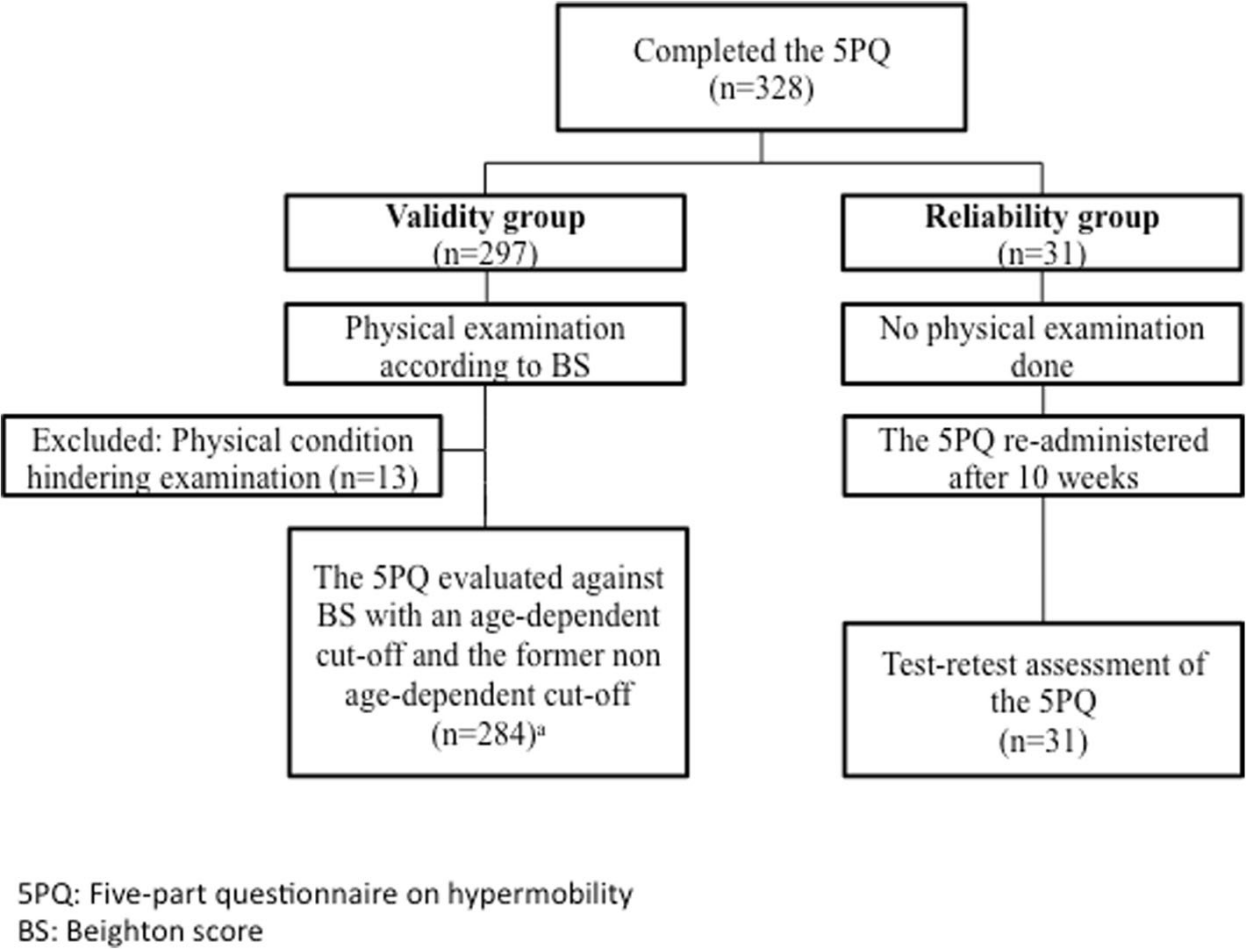
Flow of participants through the study. ^a^ The Swedish 5PQ was tested against two different criteria of the reference standard test: 1) a present Beighton Score with an age-dependent cut-off of ≥5/9 for individuals 18–50 years and ≥ 4/9 for individuals > 50 years and 2) a present BS with the traditional cut-off of ≥4/9

A total of 328 subjects were included in the study: 297 subjects in the validity group and 31 subjects in the reliability group. In the validity group, 13 subjects were excluded due to a physical condition that hindered physical examination (Fig. 1). Out of the remaining 284 subjects, 60% (*n* = 170) were women and 40% (*n* = 114) were men. The mean (±SD) age was 31.5 (12.2) years. 25% (*n* = 70) of the subjects had either one or both parents born outside of the Nordic countries: (fathers/mothers) respectively; Europe (18/15), Asia (27/30), North America (2/2), South America (5/7) and Africa (6/4), and 4 subjects had missing data on this item. 58% of the participants had on-going university studies, 1% had completed elementary school or less, 20% had completed senior high school, 7% had completed less than 3 years of university or college studies and 14% had completed 3 years or more of university or college studies. In the reliability group 68% (*n* = 21) were women, 32% (*n* = 10) were men and the mean (±SD) age was 24.8 (3.0) years. There was no missing data on the 5PQ in any of the groups. As expected, GJH was more frequent amongst women than among men (Table 2).

**Table 2:** The distribution of hypermobility according to the applied assessment method

Assessment method	Hypermobile	
	*Yes*	*No*
5PQ (Reliability group, first assessment)		
Women: n (%)	6 (28.6)	15 (71.4)
Men: n (%)	3 (30.0)	7 (70.0)
5PQ (Validity group)		
Women: n (%)	65 (38.2)	105 (61.8)
Men: n (%)	22 (19.3)	92 (80.7)
BS according to present hypermobility with an age-dependent cut-off score^a^		
Women: n (%)	20 (11.8)	150 (88.2)
Men: n (%)	3 (2.6)	11 (97.4)
BS according to present hypermobility ≥ 4/9^a^		
Women: n (%)	42 (24.7)	128 (75.3)
Men: n (%)	5 (4.4)	109 (95.6)

*5PQ* The five-part questionnaire on hypermobility

*BS* Beighton Score

^a^Two different criteria of the reference standard test were used to identify generalised joint hypermobility: 1) a present Beighton Score with an age-dependent cut-off of ≥5/9 for individuals 18–50 years and ≥ 4/9 for individuals > 50 years and 2) a present BS with the traditional cut-off of ≥4/9

### Statistics

#### Validity

When evaluated against the updated criteria of the reference test (a present BS with an age-dependent cut-off of ≥5/9 for individuals 18–50 years and ≥ 4/9 for individuals > 50 years), the Swedish 5PQ attained a sensitivity of 91% (95% CI 72, 99%) a specificity of 75% (95% CI 69, 80%) and an area under the curve of 0.87 (95% CI 0.79, 0.95). When evaluated against the traditional recommendations of the reference test (a BS according to present hypermobility, cut-off ≥4/9), the Swedish 5PQ attained a sensitivity of 72% (95% CI 57, 84%), a specificity of 78% (95% CI 72, 83%) and an area under the curve of 0.77 (95% CI 0.68, 0.85). A more detailed presentation is shown in Table 3. Post hoc analyses on additional cut-off scores on the reference test are presented in Supplementary Table S2, Additional file 2.

**Table 3:** Evaluation of validity of the Swedish 5PQ

5PQ score	Sensitivity (%) (95% CI)	Specificity (%) (95% CI)	PPV (%) (95% CI)	NPV (%) (95% CI)
BS according to present hypermobility with an age-dependent cut-off score^a^				
= 0/5	100.00 (85.18, 100.00)	0.00 (0.00, 1.40)	8.10 (8.10, 8.10)	–
≥ 1/5	95.65 (78.05, 99.89)	34.10 (28.37, 40.20)	11.34 (10.16, 12.64)	98.89 (92.85, 99.84)
≥ 2/5^b^	91.30 (71.96, 98.93)	74.71 (68.98, 79.87)	24.14 (19.96, 28.88)	98.98 (96.28, 99.73)
≥ 3/5	56.52 (34.49, 76.81)	91.57 (87.52, 94.64)	37.14 (25.67, 50.27)	95.98 (93.74, 97.45)
≥ 4/5	30.43 (13.21, 52.92)	97.32 (94.55, 98.92)	50.00 (27.75, 72.25)	94.07 (92.37, 95.42)
= 5/5	0.00 (0.00, 14.82)	99.62 (97.88, 99.99)	0	91.87 (91.82, 91.93)
BS according to present hypermobility ≥ 4/9^a^				
= 0/5	100.00 (92.45, 100.00)	0.00 (0.00, 1.54)	16.55 (16.55, 16.55)	–
≥ 1/5	85.11 (71.69, 93.80)	35.02 (28.96, 41.46)	20.62 (18.24, 23.21)	92.22 (85.42, 96.00)
≥ 2/5^b^	72.34 (57.36, 84.38)	77.64 (71.79, 82.78)	39.08 (32.31, 46.30)	93.40 (89.87, 95.76)
≥ 3/5	42.55 (28.26, 57.82)	93.67 (89.78, 96.41)	57.14 (42.45, 70.67)	89.16 (86.51, 91.33)
≥ 4/5	17.02 (7.65, 30.81)	97.47 (94.57, 99.07)	57.14 (32.66, 78.56)	85.56 (83.86, 87.10)
= 5/5	0.00 (0.00, 7.55)	99.58 (97.67, 99.99)	0	83.39 (83.28, 83.51)

*5PQ* The Five-part questionnaire on hypermobility

*BS* Beighton Score

*PPV* Positive predictive value

*NPV* Negative predictive value

^a^ The Swedish 5PQ was tested against two different criteria of the reference standard test: 1) a present Beighton Score with an age-dependent cut-off of ≥5/9 for individuals 18–50 years and ≥ 4/9 for individuals > 50 years and 2) a present BS with the traditional cut-off of ≥4/9

^b^ The recommended cut-off score for generalised joint hypermobility on the 5PQ [4]

#### Test-retest reliability

All aspects of the 5PQ showed substantial to almost perfect agreement. In the item-to-item comparison, kappa values for each item were respectively: item 1 (0.87), item 2 (0.83), item 3 (1.00), item 4 (−) and item 5 (0.71). Due to absent variance in item 4, kappa measurement of agreement was not applicable. ICC for the total-score comparison was 0.92 (95% CI 0.85, 0.96).

### Item specific analyses

The distribution of responses for each item of the 5PQ, including the odds ratio (OR) for GJH, is presented in Table 4. Item 4 "As a child or teenager did your shoulder or kneecap dislocate on more than one occasion?" fell out as non-significant to predict GJH (OR 1.56, *P* = 0.64 when tested against a present BS with an age-dependent cut-off and OR 1.09, *P* = 1.00 when tested against a BS according to present hypermobility ≥4/9).

**Table 4:** The distribution of responses to each item on the 5PQ and the OR for GJH

5PQ item number	Hypermobile		Non-hypermobile		OR for GJH (95% CI)	*P* value^a^
	Distribution of answers on the 5PQ					
	Yes	No	Yes	No		
BS according to present hypermobility with an age-dependent cut-off score^b^						
1	20	3	133	128	6.42 (1.86, 22.12)	0.001
2	16	7	59	202	7.83 (3.07, 19.92)	< 0.001
3	11	12	45	216	4.40 (1.83, 10.60)	0.001
4	2	21	15	246	1.56 (0.34, 7.30)	0.637
5	14	9	16	245	23.82 (8.96, 63.36)	< 0.001
BS according to present hypermobility ≥ 4/9^b^						
1	35	12	118	119	2.94 (1.46, 5.94)	0.002
2	29	18	46	191	6.69 (3.42, 13.08)	< 0.001
3	18	29	38	199	3.25 (1.64, 6.43)	< 0.001
4	3	44	14	223	1.09 (0.30, 3.94)	1.000
5	17	30	13	224	9.76 (4.32, 22.09)	< 0.001

*5PQ* The five-part questionnaire on hypermobility

*BS* Beighton Score

*OR* Odds ratio

*GJH* Generalised joint hypermobility

^a^ Pearson’s chi-square test or Fisher’s exact test when appropriate (2-sided)

^b^ The Swedish 5PQ was tested against two different criteria of the reference standard test: 1) a present Beighton Score with an age-dependent cut-off of ≥5/9 for individuals 18–50 years and ≥ 4/9 for individuals > 50 years, adapted to the updated recommendations [8] and 2) a present BS with the traditional cut-off of ≥4/9

## Discussion

In this study we tested validity and reliability of the 5PQ translated into Swedish on a normal adult population. We did not differentiate between asymptomatic and symptomatic GJH. When evaluated against the updated criteria of the reference standard test (a present Beighton Score with an age-dependent cut-off of ≥5/9 for individuals 18–50 years and ≥ 4/9 for individuals > 50 years) the Swedish 5PQ attained a sensitivity of 91%, a specificity of 75% and an AUC of 0.87 to identify GJH. All aspects of the 5PQ showed substantial to almost perfect test-retest reliability.

### Item specific analyses

The item 4 “As a child or teenager did your shoulder or kneecap dislocate on more than one occasion?” in the 5PQ did not predict GJH in our sample. It seems clear from Hakim and Grahame’s original 5PQ study [4] that item 4 was relevant mainly among hypermobile individuals with musculoskeletal symptoms, whereas our study assessed a non-clinical population, in which dislocation of shoulder or kneecap is rare.

### What is the intended use and clinical role of the 5PQ?

The 5PQ was initially developed as a screening tool in patients at high risk [4] but has also been shown to be helpful for identifying GJH in population based studies [5].

The Swedish 5PQ attained similar sensitivity and specificity to previous reports [4, 6]. However, the PPV was rather low. Presumably, this is a consequence of evaluating a Swedish non-clinical population and our use of the stricter criteria of the reference standard test. Together this results in a low prevalence of GJH. Accordingly, the PPV in our study would have been similar to the PPV reported in the Brazilian study [6] if the prevalence of GJH in our study group had been similar. Unfortunately these estimates were not reported in the original study [4].

In summary, our findings suggest that the 5PQ can be applied to identify GJH in studies where a physical examination is impractical, e.g. population studies. However, one needs to consider the likely prevalence of GJH in the targeted population. On the other hand, the 5PQ might actually be superior at identifying GJH than the BS, since the 5PQ inquires for a subjective general view on hypermobility, rather than focusing exclusively on 5 joints.

### The BS as reference standard

The human body has more than 230 movable or semi-movable joints [12] and the BS is limited to a few joints only. Hence, the BS will disregard hypermobility in other joints. We chose, however, to limit our study to the BS as a reference standard since this was used in the original development of the 5PQ [4].

### Prevalence rates of GJH in our study

Comparisons of prevalence must take age, gender, ethnicity, targeted population and the criterion used to define it into account, since the occurrence of GJH varies widely depending on these factors.

Considering the characteristics of the study population and the applied assessment method the Swedish 5PQ yielded a prevalence rate of GJH reasonably in accordance with previous reports. In our study the 5PQ assigned 38.2% of the females and 19.3% of the males as GJH. This is similar to the prevalence rates reported in a large (*n* = 1039) Swedish study [13] also using the Swedish 5PQ as an assessment method for GJH. One study of female twins in the UK [5] reported the prevalence rate of GJH to be around 20%, although participants aged 20–30 years (similar age to our participants) had a prevalence rate (34%), closer to our findings. In the Brazilian study [6] the 5PQ rated 43.5% of the females and 28.4% of the males as GJH. Their slightly higher prevalence of GJH may be related to ethnic origin.

However, when using the BS as an assessment method to identify GJH, there was a low prevalence amongst males in our study. Nevertheless, one study of American students attending a military academy reported even lower prevalence rates than we did [14]. Considerably higher rates of GJH were reported in another cohort of college students in the United States [15]; 13.7% of the males and 36.7% of the females were hypermobile. In their study sample, 81.3% were of North American Caucasian origin and the mean age was 20 years, hence their study is rather comparable to ours even though the age difference possibly could explain some of the difference. We have not found a comparable study describing the prevalence of GJH in Sweden. However, by interpreting the graphs presented in a Swedish study [16] that also used the BS but with a slightly different scoring system, their reported prevalence of GJH seem rather similar to ours. Assuming that ≥3/5 hypermobility features equals the cut-off of ≥5/9 that we applied, they classified approximately 7% of the males as GJH compared to our finding of approximately 4%. Possibly, divergent findings may also be related to the examiner [17].

### Translation considerations

Item 1 “Can you now (or could you ever) place your hands flat on the floor without bending your knees?” and item 2 “Can you now (or could you ever) bend your thumb to touch your forearm?” in the 5PQ are included in the BS. In our sample, 1.5% (item 1) and 23% (item 2) of the subjects who had responded negatively to the question in the 5PQ nevertheless had a present ability to do the manoeuvre in the BS assessment. The Brazilian study [6] provided an illustration of item 2 in their questionnaire. Nonetheless, 24% of the Brazilian participants who had responded negatively in the 5PQ had at least one positive score on the manoeuvre in the BS assessment. Seemingly, an illustration is insufficient to attain satisfactory understanding. If a revised version of the 5PQ is to be developed we propose the phrasing “Can you now (or could you ever) bend *either* thumb to touch your forearm? *Please try on both sides following the illustration*”.

Furthermore, item 4 “As a child or teenager did your shoulder or kneecap dislocate on more than one occasion?”, might be easily misinterpreted. During our study we gradually realised that subjects failed to acknowledge the requirement of at least two dislocations for endorsement. Therefore, we interviewed a subset of 17 subjects with a positive response on item 4 about their interpretation of the question. Seven reported that they had overlooked this requirement. We do not believe this is a translation error since the question is phrased similarly in both languages. Moreover, we doubt that a more correct interpretation of the stricter meaning actually would change the performance of the 5PQ. Nevertheless, if a revised version of the 5PQ is to be developed, we propose to phrase this item as “As a child or teenager did your shoulder or kneecap dislocate on at least two occasions? – please note that one occasion doesn’t count”, and to evaluate whether it improves the performance of the 5PQ.

### Limitations

We do acknowledge that no large scale cultural or intellectual testing was done during the translation process. Although we did not specifically make a cultural validation of the Swedish 5PQ, the performance of our version suggests an acceptable cultural adaption. The low number of individuals with an affirmative response to item 4 “As a child or teenager did your shoulder or kneecap dislocate on more than one occasion?” would have called for a rather extensive pilot group to acknowledge potential misunderstandings regarding this item. The Brazilian-Portuguese version of the 5PQ included illustrations in order to improve the interpretability. In our opinion, however, such addition would result in a revised version of the 5PQ rather than a Swedish translation of the instrument.

## Conclusions

The Swedish translation of the 5PQ demonstrated properties similar to what was reported in other linguistic versions and can be considered a valid and reliable instrument to screen for or identify GJH. Moreover, the recommended cut-off score of ≥2 for GJH on the 5PQ, was replicated in our study. A revised version of the 5PQ could include illustrations to facilitate its interpretability. Furthermore, a more detailed wording on item 2 and 4 may improve the test performance.

## Supplementary information

**Additional file 1: **Contains **Table S1**. The translation process of the Swedish 5PQ.

**Additional file 2: **Contains **Table S2**. Evaluation of validity of the Swedish 5PQ. Tested against various cut-off scores on the reference test.

**Additional file 3:** Contains The Swedish 5PQ Screen print-out version with instructions to the clinician.

## Data Availability

The datasets generated during and/or analysed during the current study are available from the corresponding author on reasonable request.

## Abbreviations

5PQ: Five-part questionnaire on hypermobility
AUC: Area under the curve
BS: Beighton Score
GJH: Generalised joint hypermobility
ICC: Intra-class correlation coefficient
NPV: Negative predictive value
OR: Odds ratios
PPV: Positive predictive value
ROC: Receiver operator characteristic
