# Supplementary material for: Self-rated joint hypermobility: the five-part questionnaire evaluated in a Swedish non-clinical adult population
Source: BMC Musculoskelet Disord. 2020 Mar 17;21:174. doi: 10.1186/s12891-020-3067-1 (PMC7079417; doi:10.1186/s12891-020-3067-1)
Supplement: Supplementary file 1 — Additional file 1: Contains Table S1. The translation process of the Swedish 5PQ. [file 12891_2020_3067_MOESM1_ESM.pdf]

Table S1. The translation process of the Swedish 5PQ.

Item	Original version of the 5PQ	Forward translation	Expert panel **(Final version)**	Back translation 1	Back translation 2
1	Can you now (or could you ever) place your hands flat on the floor without bending your knees?	Kan du nu (eller har du någonsin kunnat) placera händerna platt på golvet utan att böja knäna?	Kan du nu (eller har du någonsin kunnat) placera händerna platt på golvet utan att böja knäna?	Can you now (or have you ever been able to) place your hands flat on the floor without bending your knees?	Are you able (have you ever been able) to place your palms flat on the floor without bending your knees?
2	Can you now (or could you ever) bend your thumb to touch your forearm?	Kan du nu (eller har du någonsin kunnat) böja tummen så att den nuddar din underarm?	Kan du nu (eller har du någonsin kunnat) böja tummen så att den nuddar din underarm?	Can you now (or have you ever been able to) bend your thumb so that it touches your forearm?	Are you able to (have you ever been able to) bend your thumb so that it touches your forearm?
3	As a child did you amuse your friends by contorting your body into strange shapes OR could you do the splits?	Underhöll du dina vänner med att vrida din kropp i konstiga ställningar eller kunde du gå ner i split som barn?	Underhöll du dina vänner med att vrida din kropp i konstiga ställningar eller kunde du gå ner i split som barn?	Did you entertain your friends by twisting your body into strange positions or were you able to go down into the splits as a child?	Did you entertain your friends by twisting your body into strange positions or could you do the splits as a child?
4	As a child or teenager did your shoulder or kneecap dislocate on more than one occasion?	Har din knäskål eller axel gått ur led mer än en gång som barn eller tonåring?	Har din knäskål eller axel gått ur led mer än en gång som barn eller tonåring?	Have your kneecaps or shoulders become dislocated on more than one occasion as a child or teenager?	Have your knee-caps or shoulders been dislocated more than once as a child or teenager?
5	Do you consider yourself double-jointed?	Anser du dig själv vara extremt böjlig i lederna?	Anser du dig själv vara påtagligt överrörlig i lederna?	Do you consider yourself to be clearly hypermobile in your joints?	Do you consider yourself to have joint hypermobility?

5PQ: The five-part questionnaire on hypermobility
