# Supplementary material for: Self-rated joint hypermobility: the five-part questionnaire evaluated in a Swedish non-clinical adult population
Source: BMC Musculoskelet Disord. 2020 Mar 17;21:174. doi: 10.1186/s12891-020-3067-1 (PMC7079417; doi:10.1186/s12891-020-3067-1)
Supplement: Supplementary file 2 — Additional file 2: Contains Table S2. Evaluation of validity of the Swedish 5PQ. Tested against various cut-off scores on the reference test. [file 12891_2020_3067_MOESM2_ESM.pdf]

Table S2. Evaluation of validity of the Swedish 5PQ. Tested against various cut-off scores on the reference test.

5PQ score	Sensitivity (%) (95% CI)	Specificity (%) (95% CI)
**BS cut-off according to the current age-dependent recommendations[a]**		
≥ 1/5	95.65 (78.05, 99.89)	34.10 (28.37, 40.20)
≥ 2/5[b]	91.30 (71.96, 98.93)	74.71 (68.98, 79.87)
≥ 3/5	56.52 (34.49, 76.81)	91.57 (87.52, 94.64)
**BS cut-off one point below the recommendations[c]**		
≥ 1/5	86.00 (73.26, 94.18)	35.47 (29.35, 41.97)
≥ 2/5[b]	72.00 (57.51, 83.77)	78.21 (72.36, 83.32)
≥ 3/5	42.00 (28.19, 56.79)	94.02 (90.17, 96.69)
**BS cut-off one point above the recommendations[d]**		
≥ 1/5	100.00 (75.51, 100.00)	32.97 (27.42, 38.89)
≥ 2/5[b]	100.00 (71.51, 100.00)	72.16 (66.44, 77.39)
≥ 3/5	36.36 (10.93, 69.21)	10.26 (6.92, 14.48)

5PQ: The Five-part questionnaire on hypermobility
BS: Beighton Score
PPV: Positive predictive value
NPV: Negative predictive value

[a] A present Beighton Score with an age-dependent cut-off of ≥ 5/9 for individuals 18-50 years and ≥ 4/9 for individuals >50 years
[b] The recommended cut-off score for generalised joint hypermobility on the 5PQ[4]
[c] A present Beighton Score cut-off of ≥ 4/9 for individuals 18-50 years and ≥ 3/9 for individuals >50 years
[d] A present Beighton Score cut-off of ≥ 6/9 for individuals 18-50 years and ≥ 5/9 for individuals >50 years
