# Supplementary material for: Self-rated joint hypermobility: the five-part questionnaire evaluated in a Swedish non-clinical adult population
Source: BMC Musculoskelet Disord. 2020 Mar 17;21:174. doi: 10.1186/s12891-020-3067-1 (PMC7079417; doi:10.1186/s12891-020-3067-1)
Supplement: Supplementary file 3 — Additional file 3: Contains The Swedish 5PQ Screen print-out version with instructions to the clinician. [file 12891_2020_3067_MOESM3_ESM.pdf]

The Swedish 5PQ

Namn:	Ålder:	Kön:
Datum:	Ansvarig:	

Besvara frågorna utifrån vad som känns sant för dig. Sätt kryss i endast en ruta per fråga!

		NEJ	JA
1.	Kan du nu (eller har du någonsin kunnat) placera händerna platt på golvet utan att böja knäna?	☐	☐
2.	Kan du nu (eller har du någonsin kunnat) böja tummen så att den nuddar din underarm?	☐	☐
3.	Underhöll du dina vänner med att vrida din kropp i konstiga ställningar eller kunde du gå ner i split som barn?	☐	☐
4.	Har din knäskål eller axel gått ur led mer än en gång som barn eller tonåring?	☐	☐
5.	Anser du dig själv vara påtagligt överrörlig i lederna?	☐	☐

The Swedish 5PQ är en svensk översättning av the Hakim-Grahame five-part questionnaire on hypermobility.

Referens: Glans M, Humble M, Elwin M, Bejerot S. Self-rated joint hypermobility: The five-part questionnaire evaluated in a Swedish non-clinical adult population. BMC Musculoskelet Disord. 2020. https://doi.org/10.1186/s12891-020-3067-1.

The Swedish 5PQ

Rättning:
- Alla items poängsätts med ett poäng för “ja” och noll poäng för “nej”.
- Den sammanlagda poängen kan variera mellan 0 och 5.
- ≥ 2 poäng tyder på generalised joint hypermobility (GJH).

The Swedish 5PQ validerades på en icke-klinisk svensk vuxen population:
- Reference standard test var Beighton Score med cut-off ≥ 5/9 för individer 18-50 års ålder och ≥ 4/9 för individer >50 års ålder.
- The Swedish 5PQ uppnådde en sensitivitet på 91%, en specificitet på 75% och en area under the curve på 0.87.

The Swedish 5PQ är en svensk översättning av the Hakim-Grahame five-part questionnaire on hypermobility.

Referens: Glans M, Humble M, Elwin M, Bejerot S. Self-rated joint hypermobility: The five-part questionnaire evaluated in a Swedish non-clinical adult population. BMC Musculoskelet Disord. 2020. https://doi.org/10.1186/s12891-020-3067-1.
